# Fabrication of Porous Polyvinylidene Fluoride/Multi-Walled Carbon Nanotube Nanocomposites and Their Enhanced Thermoelectric Performance

**DOI:** 10.3390/polym10070797

**Published:** 2018-07-19

**Authors:** Fei-Peng Du, Xuan Qiao, Yan-Guang Wu, Ping Fu, Sheng-Peng Liu, Yun-Fei Zhang, Qiu-Yu Wang

**Affiliations:** School of Materials Science and Engineering, Key Laboratory of Green Chemical Process of Ministry of Education, Wuhan Institute of Technology, Wuhan 430205, China; hsdfp@163.com (F.-P.D.); 13387539176@163.com (X.Q.); wygddxyz@163.com (Y.-G.W.); fuping751128@163.com (P.F.); liuabss@163.com (S.-P.L.); WITwangqiuyu@163.com (Q.-Y.W.)

**Keywords:** thermoelectric, MWNT, PVDF, porous structure, composite technology

## Abstract

In this paper, a solvent vapor-induced phase separation (SVIPS) technique was used to create a porous structure in polyvinylidene fluoride/Multi-walled carbon nanotube (PVDF/MWNTs) composites with the aim of increasing the electrical conductivity through the incorporation of MWNTs while retaining a low thermal conductivity. By using the dimethylformamide/acetone mixture, porous networks could be generated in the PVDF/MWNTs composites upon the rapid volatilization of acetone. The electrical conductivity was gradually enhanced by the addition of MWNTs. At the same time, the thermal conductivity of the PVDF film could be retained at 0.1546 W·m^−1^·K^−1^ due to the porous structure being even by loaded with a high content of MWNTs (i.e., 15 wt.%). Thus, the Seebeck coefficient, power factor and figure of merit (*ZT*) were subsequently improved with maximum values of 324.45 μV/K, 1.679 μW·m^−1^·K^−2^, and 3.3 × 10^−3^, respectively. The microstructures, thermal properties, and thermoelectric properties of the porous PVDF/MWNTs composites were studied. It was found that the enhancement of thermoelectric properties would be attributed to the oxidation of MWNTs and the porous structure of the composites. The decrease of thermal conductivity and the increase of Seebeck coefficient were induced by the phonon scattering and energy-filtering effect. The proposed method was found to be facile and effective in creating a positive effect on the thermoelectric properties of composites.

## 1. Introduction

Converting waste heat to electricity via thermoelectric (TE) materials is an important means of developing green sustainable energy. Over a long period of time, traditional inorganic semiconductor materials, such as Bi_2_Sb_3_, Bi_2_Te_3_ and PbTe, have become well-known TE materials that have been utilized commercially for energy harvesting. TE materials are able to convert a temperature difference (*ΔT*) into an electrical voltage, in which the efficiency is reflected in the Figure of Merit, *ZT*, where *ZT* = *S*^2^*σT*/*κ, S* is the Seebeck coefficient, *σ* is the electrical conductivity, *T* is the absolute temperature, and *κ* is the thermal conductivity [1,2]. High performance TE materials are required to have a high Seebeck coefficient and electrical conductivity but low thermal conductivity. The traditional inorganic TE materials possess high *S* and *σ* due to their high density of the charge carriers. Although the high charge density also contributes to the high *κ* at the same time, their *ZT* values can still reach 1 to 2. However, some applications, such as mounting solar cells on a curved surface and the recycling of end-of-life panels, may be hindered by the high production cost and the brittleness of the substrates [2]. Recently, conducting polymer has exhibited great prospects in the thermoelectric field due to low thermal conductivity, solution processability and low preparation cost. The thermal conductivity of conducting polymer ranges from 0.02 W·m^−1^·K^−1^ and 0.6 W·m^−1^·K^−1^, which is far lower than those of conventional inorganic TE materials [3]. So far, the main conducting polymers that have been commonly used as organic thermoelectric materials include poly(3,4-ethylenedioxythiophene) (PEDOT), polypyrrole (PPy) and polyaniline (PANI) [4,5,6]. Notably, the thermoelectric performance of the polymer thermoelectric materials can be greatly enhanced though compositing with inorganic materials such as carbon nanotubes, graphene, silver nanowires, etc. [7,8,9]. 

Tuning the morphology of the polymers and their composite thermoelectric materials has been proved to be an effect way to enhance the thermoelectric performance [3]. Regulating nanostructure and creating porous structure in the polymers and their composites are extensively used to control the thermoelectric performance [10,11]. Zhang et al. have constructed a three-dimensional (3D) network morphology composed of PPy nanowires adsorbed on the graphene nanosheet surfaces, which exhibited excellent thermoelectric properties [10]. Chen et al. reported that carbon nanotubes with porous structures possess superlow thermal conductivity and high Seebeck coefficient due to the porous boundary conducting energy-filtering [11]. This energy-filtering effect strongly increases the scattering effect of low-energy carriers, while the high-energy counterparts are unaffected [12]. The use of nanoporous AgSbTe_2_ produced a large increase in the Seebeck coefficient, and subsequently resulted in an enhancement in the thermoelectric performance [13]. The increase of the Seebeck coefficient was also observed in β-FeSi_2_ compounds with micro-sized pores [14]. In addition, porous structures can reduce or restrain the thermal conductivity because of the increase of the air volume fraction in the substrates. Specifically, the use of porous silicon [15], porous Ca_9_Co_12_O_28_ [16], porous Mg_2_Si_0.5_Sn_0.5_ doped by Sb [17], porous graphene films [18] and graphene-MWNTs aerogels with porous skeleton structures [19] was investigated. Using this approach, one should be very careful to strike a balance between the thermal conductivity and electrical conductivity, because the electrical conductivity is also affected, when the scattering of the charge carriers is more pronounced in the porous structures. Thus, the electrical conductivity of the porous polymeric structures has to be increased. For instance, the introduction of nanofillers with high electrical conductivity (such as carbon nanotubes, graphene, and Bi_2_Te_3_) could increase the concentration of the charge carriers and create electrical conducting paths for the polymer matrix [20,21,22]. However, the thermal conductivity is also increased. A balance between the thermal conductivity and electrical conductivity becomes an important issue for improving the thermoelectric properties of polymer composites. Carbon nanotubes (CNTs) are commonly used as the fillers because CNTs possess Seebeck coefficients of 20 μV/K–80 μV/K, excellent electrical conductivity (10^2^–10^8^ S/m), high corresponsive power factor (*S*^2^*σ*) [23], and high thermal conductivity, i.e., 3000 W·m^−1^·K^−1^ for MWNTs and 6000 W·m^−1^·K^−1^ for single-walled carbon nanotubes (SWNTs) [24,25]. Studies have shown that porous polystyrene/CNT bundled composites exhibit high electrical conductivity (125,000 S/m) but lower thermal conductivity (0.3 W·m^−1^·K^−1^) with a *ZT* value of ~0.41, where the low thermal conductivity was mainly attributed to the large number of voids in the composites [26,27]. Chen et al. coated CNTs with polyaniline (PANi) and fabricated porous structures with a three-dimensional (3-D) network of CNTs [10], leading to a drastic reduction of the thermal conductivity of the composites to 0.035 W·m^−1^·K^−1^. Porous MWNTs/PANI hybrids also exhibited an enhanced thermoelectric performance via the energy-filtering effect and phonon scattering effect [11].

Owing to the low thermal conductivity, excellent processability and thermal stability, polyvinylidene fluoride (PVDF) has been considered as a potential thermoelectric matrix for developing high-performance and cheap thermoelectric devices [28]. Carroll and co-workers designed a multi-layer CNT/PVDF-PVDF structure for the fabrication of thermoelectric fabrics, and also assembled tellurium nanorods in a PVDF matrix to balance the flexibility and the thermoelectric properties of the composites. The prepared thermoelectric fabric, which remained flexible and lightweight, is excellent for portable lightweight electronics [29,30]. In addition, N-type flexible thermoelectric fabrics based on Bi_2_Se_3_ nanoplate/PVDF composites were prepared and the composites exhibited a high Seebeck coefficient (−80 μV/K), high electrical conductivity (5100 S/m), low thermal conductivity (0.42 W·m^−1^·K^−1^) and an estimated *ZT* value of 0.02 [28]. Although the electrical conductivity of PVDF composites was improved by adding carbon nanomaterials [31,32], their high thermal conductivity still restrained the thermoelectric efficiency [33].

In our study, a facile method is proposed to fabricate a porous PVDF structure filled with MWNTs via solvent vapor-induced phase separation (SVIPS). The dispersion of MWNTs in the PVDF matrix was facilitated through the oxidation of the MWNTs surface. Using this method, an improvement in the thermoelectric properties was successfully demonstrated in the porous PVDF/MWNTs composites.

## 2. Experimentation

### 2.1. Materials

MWNTs were purchased from the Shenzhen Nanotech Port Co., Ltd. (Shenzhen, China) with more than 97% of purity, 5–15 μm in length and 40–60 nm in diameter. PVDF powders (HSV900) were purchased from the Arkema Co. (Colombes, France) with the molecular weight (Mw) of 1 million. Other reagents (Analytical reagent) were purchased from the Sinopharm Chemical reagent Co., Ltd. (Shanghai, China) and used as received.

### 2.2. Preparation of Porous PVDF/MWNTs Composites

Porous PVDF-based composites were fabricated to obtain porous materials via solvent vapor-induced phase separation (SVIPS) [34]. DMF, with its high boiling point, is an excellent solvent for PVDF. To enhance the evaporation rate and foster the formation of porous PVDF, acetone with a low boiling point was mixed with DMF. Firstly, MWNTs were acidically treated with a mixture of concentrated sulfuric acid and concentrated nitric acid (*V*/*V* = 1:3) for 6 h at 100 °C to obtain carboxylic acid groups and hydroxyl groups containing surfaces as described in [35]. The 10 wt.% PVDF solution was obtained by dissolving the PVDF into the mixed solvent of DMF and acetone (*V*/*V* = 6:4). Different amounts of MWNTs were added into the PVDF solution under ultrasound treatment for 1 h, followed by magnetic stirring for 4 h. Finally, the solutions were cast on a glass plate to form a uniform membrane under natural drying for 24 h and then dried in an oven at 80 °C for 12 h. The resulting composites were cut and trimmed into rectangular strips of 10 mm wide, 100 mm long and 100 μm thick.

### 2.3. Characterization

The morphology and microstructures of PVDF/MWNT composites were characterized by scanning electron microscopy (SEM) (Phillips XL30, FEI Company, Hillsboro, OR, USA). The samples were firstly treated via a liquid nitrogen cryogenic-fractured method to obtain fracture cross-sections. The thermal behavior and crystallinity of the membranes were determined through differential scanning calorimetry (DSC) (TA Q2000, New Castle, DE, USA equipped with TA Universal Analysis software). A sample of about 3 mg was placed in a 40 μL aluminum crucible and heated from 30 to 220 °C at a rate of 10 °C/min. The heat flow was recorded and then the crystallinity (*χ*) was calculated by using the equation (*χ* = *ΔH_m_*/*ΔH_100%_*). *ΔH_100%_* represents the melting enthalpy of the pure crystalline PVDF, which was reported to be 104.9 J/g [36]. The electrical conductivity and Seebeck coefficient were measured using a Thermoelectric Parameter Test system (Namicro-III, Wuhan Schwab Instruments, Wuhan, China). For measuring the Seebeck coefficient, all the samples had similar dimensions of 14.50 mm × 14.50 mm × 40 μm [length (l) × width (w) × thickness (d)] and five samples were characterized to obtain the average value for each PVDF/MWNTs composites. The temperature gradient of collection points was set from 0.3 to 3.5 °C along the longitudinal direction of samples. The thermal conductivity was measured via a quick thermal conductivity meter (QTM-500, KEM, Kyoto, Japan). The power factor was obtained via the formula (*P* = *S*^2^*σ*), and the figure of merit (*ZT*) at 25 °C was obtained by using the formula (*ZT* = *S*^2^*σT*/*k*).

## 3. Results and Discussion

**Figure 1:**
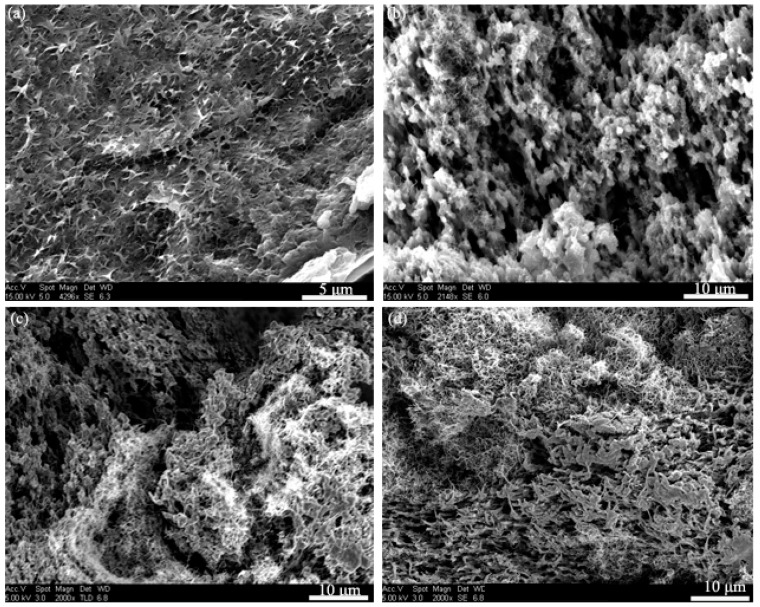
The cross-section microstructure of PVDF/MWNTs composites with (**a**) 7 wt.% MWNTs; (**b**) 15 wt.% MWNTs; (**c**) 25 wt.% MWNTs and (**d**) 35 wt.% MWNTs.

Figure 1 shows the microstructures for the fractured cross-sections of the PVDF/MWNTs composites with different MWNT content. The SEM images clearly depict the distribution of MWNTs as an inorganic phase and PVDF as an organic phase in the composites. The porous structure of the pure PVDF (Figure 1a) was created due to the use of DMF/acetone followed by the solvent-induced phase separation process. With the addition of 7 wt.% MWNTs, the PVDF formed a coral-reef-like structure that showed particles of ca. 2 μm in diameter (Figure 1b). It is clearly seen that the PVDF particles contact each other to form a continuous phase for the composite with 25 wt.% MWNTs. For Figure 1c, MWNTs and PVDF formed a double continuous phase where the 3-D networks of MWNTs were formed via entangling. With further increase in the MWNTs content to 35 wt.%, there is no significant change in morphology, as shown in Figure 1d. As shown in Figure 1, many voids and pores were created by SVIPS in all samples. It is well-known that DMF is an excellent solvent to dissolve PVDF for the dispersion of MWNTs, while acetone performs oppositely. As reported by California et al., the porosity of PVDF membranes can be controlled by adjusting the polymer/solvent concentrations and the solvent evaporation kinetics [34]. For the uniform PVDF/MWNTs solution, MWNTs have a strong nucleation effect which can facilitate polymer precipitation during the solvent evaporation process. The phase separation and volume contraction in the micro-region induced the porous structures of the composites. Moreover, CNTs with a high aspect ratio (length vs. width) increase the chance to entangle with each other, leading to the generation of porous structures with a 3D network of CNTs in the PVDF/MWNTs composites. As observed, a continuous polymer phase was formed for pure PVDF and PVDF composites at a low MWNT content, and the MWNT fillers were embedded into the PVDF phase, as shown in Figure 1a,b. When the content of the MWNTs was increased, nanotubes entangled with each other and a network of MWNTs was formed in the PVDF composites (Figure 1c,d).

**Figure 2:**
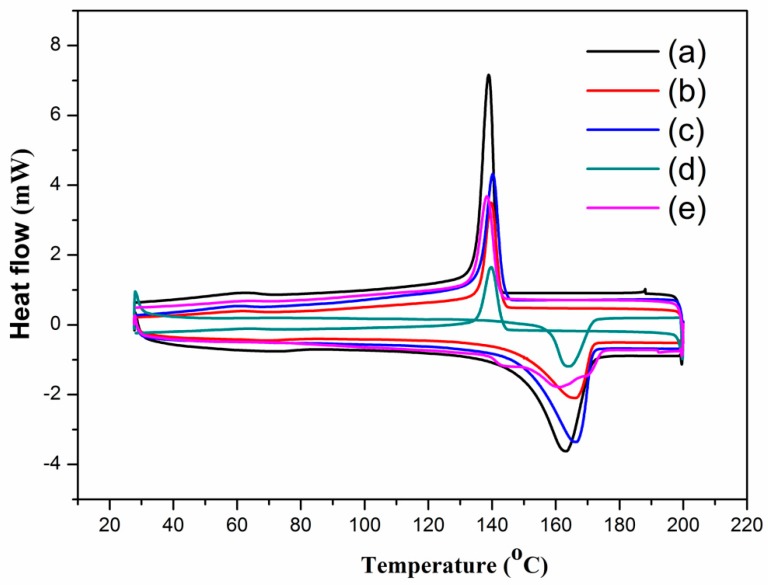
DSC thermograms of PVDF/MWNTs composites with (**a**) pure PVDF; (**b**) 7 wt.% MWNTs, (**c**) 15 wt.% MWNTs; (**d**) 25 wt.% MWNTs and (**e**) 35 wt.% MWNTs.

The effect of MWNTs on the thermal properties (such as melting temperature and melting enthalpy) of the PVDF composites was investigated by DSC. The results are shown in Figure 2, where the melting temperature slightly changed according to the amount of MWNTs in the composites. The melting temperature of the PVDF increased from 163 °C to 166.5 °C when the MWNT content was increased from 0 to 15 wt.%. Some of the important parameters are listed in Table 1, for example, the melting enthalpy was increased when the amount of MWNTs content increased from 0 to 15 wt.%, indicating that the PVDF loaded with different amounts of MWNTs has a higher structural integrity than that of pure PVDF. In addition, a higher crystallization temperature was obtained in the PVDF/MWNT samples as compared with the pure PVDF, as shown in the cooling curves of the PVDF/MWNTs composites with an MWNT content of less than 15 wt.%. Our results showed good agreement to those in [37,38] that the PVDF with a small amount of MWNTs is relatively easy crystallize. Therefore, the MWNTs phases dispersed within the PVDF matrix can promote heterogeneous nucleation during the formation of the PVDF/MWNTs composites. However, when the MWNT content is higher than 15 wt.%, the peaks of the melting temperature and the crystallization temperature of PVDF/MWNTs decrease. This is because a high content of MWNTs forms a continuous phase in the PVDF/MWNTs composites (as shown in Figure 1c,d), thus impeding the mobility of the PVDF polymer chains.

**Figure 3:**
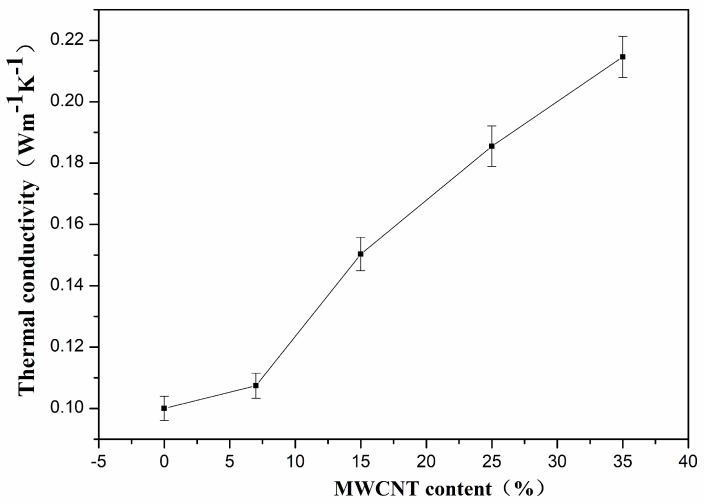
The thermal conductivity of PVDF/MWNTs composites with different content of MWNTs at room temperature.

Figure 3 shows the thermal conductivity of PVDF composites with different MWNT content at room temperature. When the MWNT content was increased from 0 wt.% to 35 wt.%, the thermal conductivity increased from 0.1012 W·m^−1^·K^−1^ to 0.2149 W·m^−1^·K^−1^. The thermal conductivity of the PVDF matrix was enhanced by ~100% through incorporation of MWNTs. However, it is very important that the obtained conductivities are still very low, so the composites can be used as a thermoelectric material [33,39]. As reported in the literature [33,40], the thermal conductivity of pure PVDF and PVDF/CNTs composites can be varied from 0.19 W·m^−1^·K^−1^ to 0.5 W·m^−1^·K^−1^. The low thermal conductivity values obtained in this work can be attributed to the porous structures in the polymer matrix as shown in Figure 1. The voids in the composites are filled with air and there are two types of interfaces in the composites: the MWNTs/polymer interfaces and the air/composites interface. As reported, the phonons mismatch and poor interfacial bonding between the MWNTs and polymer resulted in severe phonon scattering at the interface so that the thermal conductivities of polymer/MWNT composites are far lower than the theoretical values [9,33]. Yu et al. reported that the porous structure could retain the low thermal conductivity of polymer composites, even when loaded with a high content of CNTs. The phonon scattering at the interface hindered the phonon propagation that resulted in poor heat conduction [41]. Using this approach, Suemori et al. demonstrated a low thermal conductivity of polystyrene with 75 wt.% CNTs due to the plentiful amount of voids in the composite [27].

**Figure 4:**
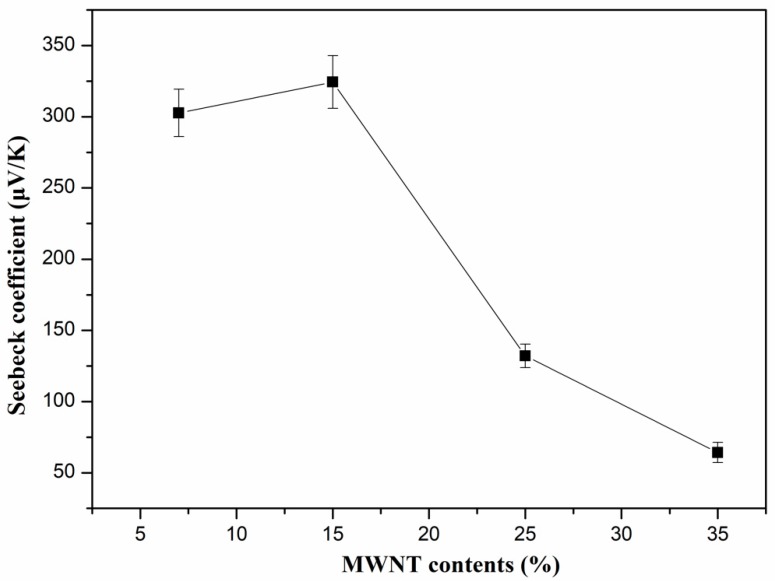
Seebeck coefficient of PVDF/MWNTs composites with different content of MWNTs at room temperature.

Figure 4 shows the Seebeck coefficient of PVDF/MWNTs composites with different CNT content at room temperature (298.15 K). The Seebeck coefficient of the PVDF composites varied with the MWNT content. When the MWNT content was increased from 7 to 15, 25, and 35 wt.%, the Seebeck coefficient changed from 302.7 to 324.45, 132.14 and 64.21 μV/K, respectively. As mentioned in the literature, the Seebeck coefficient was affected by the charge carrier concentration and the high oxygen amount of the carboxylated MWNTs acted as a *p*-dopant, facilitating the electron withdrawal from the MWNTs backbone and resulting in an increase in the Seebeck coefficient [42,43,44]. Therefore, the PVDF/MWNTs composite with 15 wt.% of MWNTs exhibits high Seebeck coefficient of about 324.45 μV/K at room temperature. However, when the MWNTs became a continuous phase after their content reached 15 wt.% or above, the high carrier concentration led to a decrease in the Seebeck coefficient, especially for the 35 wt.% sample [1]. In addition, the porous structure can also lead to the improvement of the Seebeck coefficient due to the boundary conducting energy-filtering effect, resulting in a stronger scattering of low-energy carriers while the high-energy ones are unaffected [12].

**Figure 5:**
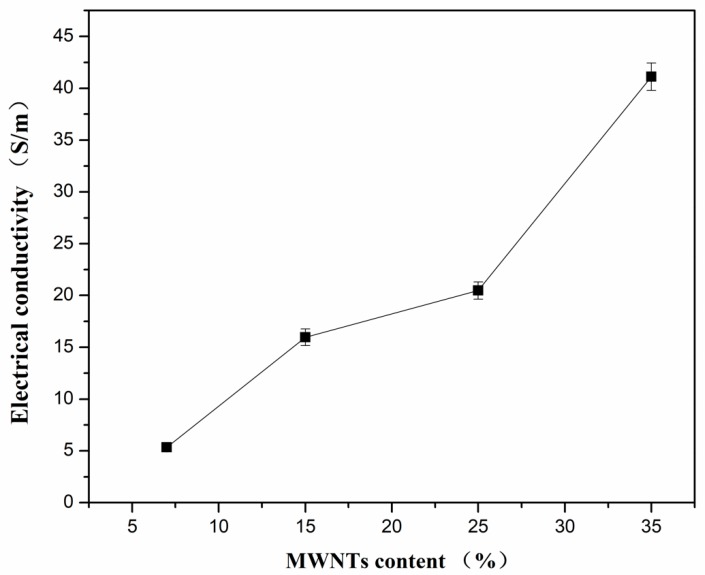
Electrical conductivity of PVDF composites with different content of MWNTs at room temperature.

The electrical conductivity of the PVDF composites with different MWNT content at room temperature is depicted in Figure 5. As described above, materials with a low carrier concentration have a large Seebeck coefficient when the content of MWNTs is lower than 15 wt.%. However, a low carrier concentration results in low electrical conductivity. When the content of MWNTs is below 15 wt.%, the PVDF acts as a continuous phase and MWNTs as a dispersive phase (as seen in Figure 1a,b). The electrically conductive paths were formed with a low content of MWNTs, leading to the increase of electrical conductivity [10,45]. The variation of electrical conductivity can be attributed to the microstructural phase transition induced by the relative content of MWNTs and PVDF. When the MWNT content increases, the electrical conductivity of the PVDF composites increases gradually, with a value of 41 S/m for the composite films with 35 wt.% of MWNTs.

**Figure 6:**
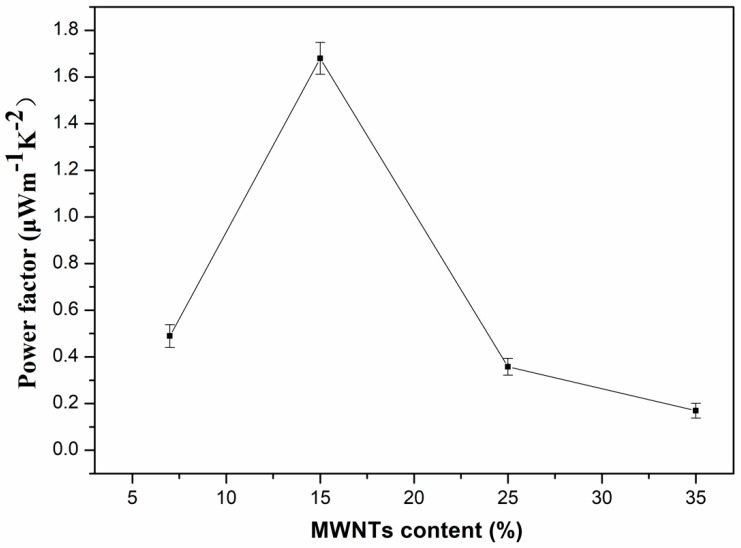
Power factor of PVDF composites with different content of MWNTs at room temperature.

The power factor (*S*^2^*σ*) was calculated in order to investigate the energy transfer capability of the PVDF composites based on the Seebeck coefficient and electrical conductivity. Figure 6 shows the power factor of PVDF composites with different MWNT content at room temperature. The power factor increases sharply until the MWNT content reaches 15 wt.% with a maximum value of 1.679 μW·m^−1^·K^−2^. The maximum value obtained in our study is higher than that of the MWNTs network/PANI with 30 wt.% MWNTs (~0.5 μW·m^−1^·K^−2^) [46], and is close to those of the CNT network/PANI composite with 44 wt.% CNTs (~2.19 μW·m^−1^·K^−2^) and MWNTs/polypyrrole composites with 20 wt.% MWNTs (~2.079 μW·m^−1^·K^−2^) [10,47]. The variation of power factor was positively correlated with the Seebeck coefficient and electrical conductivity, and the thermoelectric properties were greatly affected by the distribution of the inorganic phase (MWNTs) and organic phase (PVDF). When the PVDF was in a continuous phase and the low content of MWNTs acted as the dispersive phase, the power factor was high, because of the high Seebeck coefficient and electrical conductivity of the composites. Inversely, the power factor decreased sharply due to the relatively low Seebeck coefficient when MWNTs became the continuous phase.

**Figure 7:**
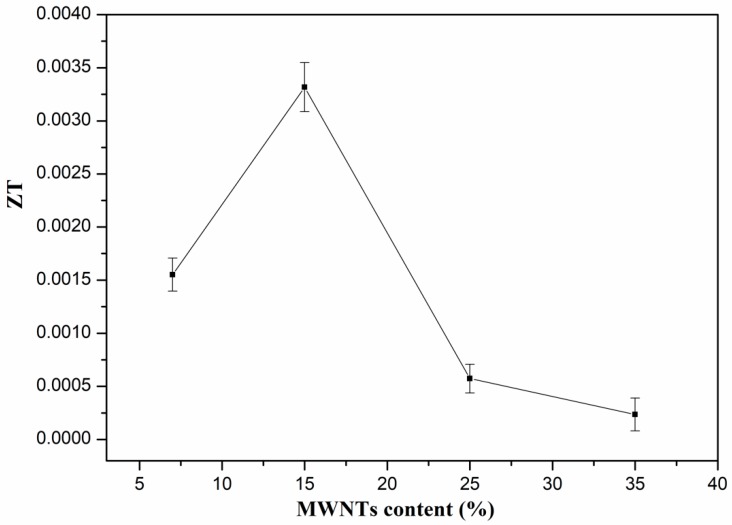
ZT values of PVDF composites with different content of MWNTs at room temperature.

The *ZT* values of the PVDF composites are shown in Figure 7. The variation of *ZT* is similar to the trend of the power factor. The *ZT* value increases with the content of MWNTs from 7 wt.% to 15 wt.%, while it decreases sharply beyond 15 wt.%. The maximum *ZT* value is 3.3 × 10^−3^. The *ZT* value is higher than that of MWNTs network/PC composites with 2.5 wt.% MWNTs [44]. Table 2 shows a comparison of the thermoelectric properties obtained from this work as compared with the results of some typical polymer/CNTs composites. It is found that the *ZT* values of PVDF/MWNTs composites prepared in this work are higher than those of the PVDF/MWNTs composites with 8 wt.% MWNTs reported in [48]. It further confirms that the PVDF/MWNTs composites with porous structures have relative high *ZT* values, and exhibit high potential for producing flexible thermoelectric devices.

In general, porous PVDF/MWNTs composites can be successfully fabricated through the oxidation of MWNTs and by using the SVIPS technique. The PVDF/MWNTs composites and the preparation method demonstrated in this study are environmentally friendly, cost effective, and easy for large scale production. The prepared PVDF/MWNTs composites exhibited low thermal conductivity, high Seebeck coefficient and relatively high power factor, demonstrating a great potential in the preparation of lightweight and cheap thermoelectric devices, such as wearable thermoelectric devices. The porous structures can balance the thermal conductivity and electrical conductivity of the composites. Therefore, downstream studies can focus on the device fabrication methods such as the electrospinning technique to make the composites with optimal architecture and to harvest energy from waste heat. 

## 4. Conclusions

In this study, porous PVDF/MWNTs composites were prepared by composite technology enhanced with the SVIPS technique. The thermal conductivity of PVDF composites remained at a very low level (0.2149 W·m^−1^·K^−1^) even when the MWNT content reached 35 wt.%. The electrical conductivity and the Seebeck coefficient were enhanced through incorporation of MWNTs into the PVDF. When the content of MWNTs was 15 wt.%, the PVDF/MWNTs composites exhibited the highest Seebeck coefficient, power factor and *ZT* values of 324.45 μV/K, 1.679 μW·m^−1^·K^−2^ and 3.3 × 10^−3^ at room temperature, respectively. This study demonstrates a facile method to prepare a porous structured composite film with an improvement in thermoelectric properties, which can be beneficial for the production of low cost, nontoxic and wearable thermoelectric devices.

## Figures and Tables

**Table 1:** Area of the melting peak of the PVDF/MWNTs composites.

Sample	Melting Enthalpy (J/g)	Crystallinity (%)
Pure PVDF	47.5	45.3
PVDF/7 wt.% MWNTs	58.8	56.1
PVDF/15 wt.% MWNTs	59.5	56.7
PVDF/25 wt.% MWNTs	41.0	39.1
PVDF/35 wt.% MWNTs	39.4	37.6

**Table 2:** A summary of the maximum thermoelectric performance for some typical polymer/MWNTs composites.

Polymer	Nanofiller	Preparation Method	Electrical Conductivity (S/m)	Seebeck Coefficient (μV/K)	Power Factor (μW·m^−1^·K^−2^)	Thermal Conductivity W·m^−1^·K^−1^	*ZT* (T)	Ref.
Polyaniline	Porous MWNT (44 wt.%)	Doping/Solution mixing	4035	23.3	2.19	0.035	1.9 × 10^−2^ (298 K)	[11]
Polyaniline	MWNT sheets (80 wt.%)	In situ chemical polymerization	~11,300	22.3	~5.6	~0.5	~3.4 × 10^−3^ (300 K)	[46]
Polycarbonate	MWNT (2.5 wt.%)	Melt extrusion	0.01	11.3	~1.3 × 10^−6^	0.29 ± 0.01	~1.3 × 10^−9^ (298 K)	[44]
PVDF	MWNTs (8 wt.%)	Melt extrusion	~0.01 (solid) <0.01 (foam)	~12 (solid) ~8 (foam)	~1.4 × 10^−6^ 6.4 × 10^−7^	~0.57 (solid) ~0.09 (foam)	~7.5 × 10^−10^ 2.1 × 10^−9^ (298 K)	[48]
Polypyrrole	MWNT (20 wt.%)	In situ chemical polymerization	~3300	~25.1	2.079	-----	----- (295 K)	[47]
polythiophene	MWNT (80 wt.%)	Ball milling/Solution mixing/In situ composite	~2100	27.2	~1.6 × 10^−2^	0.77	6.3 × 10^−6^ (303 K)	[49]
PVDF	MWNTs (15 wt.%)	Mixed solvent/phase separation	~16	324.45	1.679	0.15	3.3 × 10^−3^ (298 K)	This paper
